# Assessment of health-related quality of life in patients receiving stem cell therapy for end-stage liver disease: an Egyptian study

**DOI:** 10.1186/scrt140

**Published:** 2012-12-03

**Authors:** Hosny Salama, Abdel-Rahamn N Zekri, Rasha Ahmed, Iman Medhat, El Sayed Abdallah, Tarneem Darwish, Ola S Ahmed, Abeer Bahnassy

**Affiliations:** 1 Endemic Medicine Department, Faculty of Medicine, Cairo University, Al-Saray Street, El Manial, 11956, Cairo, Egypt; 2 Virology and Immunology Unit, Cancer Biology Department, National Cancer Institute, Cairo University, Fom El Khalig, 11796, Cairo, Egypt; 3 Internal Medicine Department, Faculty of Medicine, Cairo University, Al-Saray Street, El Manial, 11956, Cairo, Egypt; 4 Biomedical Informatics & Biostatistics, Faculty of Medicine, Cairo University, Al-Saray Street, El Manial, 11956, Cairo, Egypt; 5 Pathology Departments, National Cancer Institute, Cairo University, Fom El Khalig, 11796, Cairo, Egypt

## Abstract

**Introduction:**

This prospective cohort study aimed to assess the influence of stem cell therapy (SCT) on health-related quality of life (HRQOL) by using the SF-36 v2 and to elucidate the influence of objective clinical variables on subjective HRQOL.

**Methods:**

The study included 100 chronic liver disease patients (50 received SCT, and 50 received supportive medical treatment (SMT)). Both groups completed a modified SF-36 v2 form before therapy and at 1-, 3-, 6-, and 12-month intervals. Fifty healthy Egyptian volunteers were enrolled in the study and completed the SF-36 v2 form once.

**Results:**

Both SCT and SMT groups showed significantly lower pretherapy SF 36 v2 scores compared with healthy volunteers. In SCT-treated patients, limited complications were encountered (SF-36 v2 scores showed significant improvement in all domains throughout the follow-up period) compared with the deterioration shown by SMT patients after therapy. A significant association was detected between SF-36 v2 scores and laboratory data in SCT patients during the first month after therapy. The grade of ascites improved during the follow-up in SCT compared with SMT patients. The mean survival time was 277.56 days (95% CI, 246.217 to 308.903) for SMT and 359.300 days (95% CI, 353.022 to 365.578) for SCT patients (log rank, 0.00). Stem cell-treated patients showed no malignancies.

**Conclusions:**

SCT positively affects health-related quality of life in cirrhosis patients. The survival rate was significantly improved after SCT.

## Introduction

Chronic liver disease (CLD) leads annually to disability of hundreds of thousands of patients worldwide. Cell therapy with embryonic fetal, mononuclear, mesenchymal stromal cells is the most advanced front of modern biotechnology and medicine [1]. An important part of treatment is to improve the health-related quality of life (HRQoL) and to support the ability to cope with stressors related to disease [2]. The Short Form-36 (SF-36) health survey is a generic health-status measurement consisting of 36 items in eight domains; these eight scales are then combined to form two distinct higher-ordered clusters, the physical and mental health components, which have demonstrated good reliability and validity in chronic disease populations, including in patients with chronic liver diseases. In clinical practice, HRQoL prediction from objective variables is necessary and may be useful. For that purpose, the relation between subjective HRQoL scores and objective clinical variables, such as the presence of ascites, the status of hepatocellular carcinoma (HCC), and laboratory data, should be further analyzed [3].

Many health surveys and scoring systems are available, but not enough published data are available worldwide on the quality-of-life assessment for the patients receiving stem cell therapy [4]. We studied the effect of stem cell therapy on HRQoL by using the SF-36 v2.

## Materials and methods

### Study objectives

We conducted this study to elucidate the influence of stem cell therapy on HRQoL by using the SF-36 v2 in patients with end-stage liver disease, and to detect the influence of the objective clinical variables on the subjective HRQoL scores.

### Methods and study design

This prospective cohort study was conducted after being approved by the local Ethics Committees and Health Authorities as a continuation of the workup of our previously published study [5]. A written informed consent was obtained from all patients and normal control subjects before enrolment in the study, and the ethical committee of the National Cancer Institute and Kasr Alini hospital, Cairo University, approved the protocol, which was in accordance with the ethical guidelines of the Declaration of Helsinki (Organization No. IORG0003381; IRB 0004025, Valid to 04 Dec, 2013).

We modified the SF-36 v2 survey questionnaire, which was originally in the English language. It was translated into Arabic. Some questions were repeated with the same meaning, especially in the Arabic formula, so the survey was slightly modified before its distribution to be easily understood. This allowed more accurate and specific responses from the Egyptian population. We met our candidates personally or talked to them by telephone to participate in the study; this step agrees with that made in other studies [6,7].

The questionnaire was carefully explained to the candidates; the period of each survey was from 10 to15 minutes. The questionnaire was used to assess liver disease and the effect of stem cell transplantation on the quality of life of patients. This agrees with that done in the studies of El Garem *et al. *[8], El-Serafy *et al. *[6], and Saly *et al. *[7] All patients were asked to complete the survey before and after therapy at a fixed time points, together with laboratory investigations at the same time; this agrees with what was done by El Garem *et al. *[8].

### Patients' demographics

#### Patient selection and data collection

Patients of our study (*n *= 100) were selected from those with advanced posthepatitis C virus liver cirrhosis and WHO performance score of less than 2, attending the hepatology clinic Kasr Alini Hospital, Cairo University, (a nonprofit hospital), during the period from June 2009 to May 2010, to receive standard care according to their needs, but with no great improvement in their biochemical profile or the ascites status on follow-up. Patients who matched the inclusion criteria for stem cell therapy were assigned to SCT group I (*n *= 50) to receive SCT only, and the other patients who had not received SCT either because they refused or they were uncooperative were assigned to the SMT group II (*n *= 50) to receive the standard care according to their needs, including human albumin transfusion, fresh plasma, and vitamin K, according to the patients' needs, and were followed up through our study together with those of the SCT group (I). A group of apparently healthy volunteers free from any chronic liver disease (*n *= 50) were included in our study to provide the normal range of scores of the SF-36 v2 questionnaire of the healthy Egyptian population to be compared with the studied group of patients. Uncooperative patients and patients with any other chronic diseases, HCC, or severe bleeding tendency, were excluded from our study.

After the patient's informed-consent form was signed, the following data were collected from patients involved in this study. Demographic features of the patients including age and sex, detailed medical history, findings at physical examination, laboratory tests: (complete blood count, liver-function test, aspartate aminotransferase (AST), alanine transaminase (ALT), alkaline phosphatase (ALP), serum bilirubin, prothrombin concentration, INR, and serum creatinine. Conventional abdominal ultrasonography was performed.

All patients involved in our study were asked to complete the SF-36 questionnaire before therapy, and at four posttherapy time points (1, 3, 6 months, and at 1 year), allowing changes in individual responses over time as well as group responses at specific time points to be analyzed and compared between both groups, together with performing all the formerly mentioned investigations at the same time.

### Stem cell therapy

#### Clinical protocol

##### Days 1 to 5

Patients received a daily subcutaneous injection of 300 g of G-CSF (Neupogen; Roche Pharmaceutical) (Made in Switzerland by F. Hoffmann-La Roche Ltd, Basel) for 5 days to increase the numbers of circulating hematopoietic stem cells.

##### Day 6

Patients underwent a leukapheresis procedure in the Department of Hematology, National Cancer Institute. After the collection, the cells were transferred to the stem cell laboratory for immunomagnetic separation of the CD34^+ ^stem cell population. The CD34^+ ^cells were placed in culture for amplification and differentiation from day 6 to day 13.

###### Laboratory methods

####### Cell source, isolation, and cultivation

G-CSF-mobilized peripheral blood (200 ml) was obtained with leukapheresis, and CD34^+ ^cells were isolated from that blood by adding one volume of blood to five to 10 volumes of the lysis buffer (155 m*M *NH_4_Cl, 10 m*M *KHCO_3_, and 0.1 m*M *EDTA) for 30 minutes at 4°C to remove RBCs and to separate mononuclear cells (MNCs). The collected MNCs were washed twice with buffer (phosphate-buffered saline (PBS), pH 7.4, supplemented with 0.5% bovine serum albumin and 5 m*M *EDTA), and centrifuged at 1,500 rpm for 10 minutes. The MNCs were counted, adjusted to 2 × 10^9^/ml, and centrifuged. CD34^+ ^cells were isolated by using the CD34^+ ^cell-selection kit (CliniMacs, Germany). The cells were cultured in MEM/Ham F12 (1:1) containing 10% BSA, 1% penicillin/streptomycin, 1 ng/ml GM-CSF, and growth factor (multiplication stimulating factor: liver extract, 20 mg/ml; Sigma Lot 35F-0165) and incubated for 5 to 7 days at 37°C in 5% CO_2_. Morphology of the cultured CD34^+ ^cells was assayed with phase-contrast microscopy.

##### Day 13

Patients were admitted for collection of blood for CBC and liver-function tests. The patients were infused with approximately 1 billion expanded CD34^+ ^stem cells suspended in normal physiologic saline into either the portal vein, if hepatopedal flow, or hepatic artery, if hepatofugal flow, under ultrasound or CT guidance by using complete aseptic precautions with an 18-G needle and the 3.5-MHz probe of the ultrasonography machine. The technique used is described in detail in our previous publication [5].

### Statistical methods

SF-v2 questionnaire scores were calculated for each patient in the three groups, and patient data were analyzed by using SPSS 17.0 for Windows 7. Quantitative variables were expressed as mean ± SD (standard deviation) and compared by using Student *t *test, Mann-Whitney *U*, and Kruskal-Wallis *H *test, when appropriate, whereas qualitative variables were expressed in terms of numbers (frequency) and percentage, compared between groups with the χ^2 ^test. A general linear model with repeated measures was performed with pairwise comparisons based on estimated marginal means. Correlation coefficient was calculated by using Spearman rho and Pearson correlation, as appropriate. A Kaplan-Meier curve was constructed to calculate and compare the survival time for each group. A *P *value is considered significant when < 0.05.

## Results

**Figure 1:**
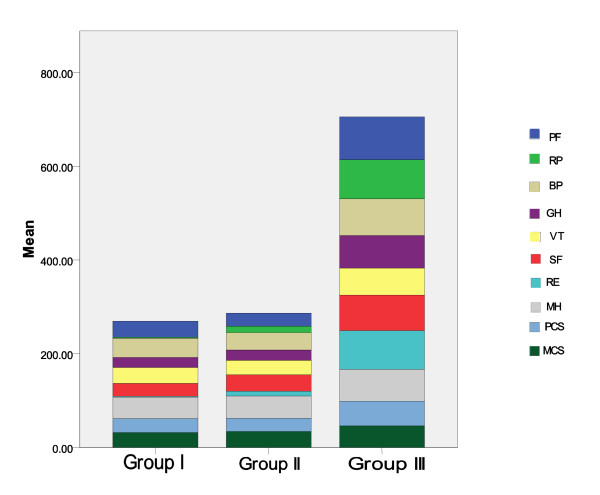
**The eight SF-36 v2 domains scores among the studied groups of patients**. BP, bodily pain; group I, SCT group; group II, SMT group; group III, healthy volunteers group; GH, general health; MCS, mental component summary; MH, mental health; PCS, physical component summary; PF, physical functioning; RE, role emotional; RP, role physical; SF, social functioning; VT, vitality.

Analyzing the data of the studied groups, we found statistically significant differences regarding age and sex distribution, residency, occupation, and marital status; their demographic and clinical data are represented in Table 1. The scores of all SF-36 questionnaire domains were calculated and compared between the three studied groups, and the results are represented in Figure 1 and Table 2.

**Table 1:** Demographic and clinical data of the studied groups

	SCT Group I (*n *= 50)	SMT Group II (*n *= 50)	Healthy volunteers Group III (*n *= 50)	*P *value
Age (mean ± SD)	51.84 ± 7.57	50.36 ± 7.01	46.40 ± 10.77	> 0.05

Gender (%)				
Male	42 (84%)	40 (80%)	40 (80%)	> 0.05
Female	8 (16%)	10 (20%)	10 (20%)	

Residency (%)				
Urban	38 (76%)	39 (78%)	40 (80%)	> 0.05
Rural	12 (24%)	11 (22%)	10 (20%)	

Occupation (%)				
Administrative, managerial, and professional	23 (46%)	23 (46%)	26 (52%)	> 0.05
Other occupations	27 (54%)	27 (54%)	24 (48%)	

Marital status (%)				
Married	50 (100%)	50 (100%)	46 (92%)	> 0.05
Unmarried	0 (0)	0 (0)	6 (8%)	

Jaundice	24	22		> 0.05

History of encephalopathy	12	20		> 0.05

Weight loss	32	29		> 0.05

Peripheral erythema	42	39		> 0.05

Lower-limb edema	32	33		> 0.05

**Table 2:** The *P *value of comparisons of the SF-v2 domain scores among the studied groups

SF-v2	*P *value SCT group (I) versus healthy volunteers group (III)	*P *value SCT group (I) versus SMT group II
PF	0.00	0.04

RP	0.00	0.001

BP	0.00	0.19

GH	0.00	0.81

VT	0.00	0.14

SF	0.00	0.01

RE	0.00	0.01

MH	0.00	0.28

PCS	0.00	0.17

MCS	0.00	0.004

BP, bodily pain; GH, general health; MCS, mental component summary; MH, mental health; PCS, physical component summary; PF, physical functioning; RE, role emotional; RP, role physical; SF, social functioning; VT, vitality.

**Figure 2:**
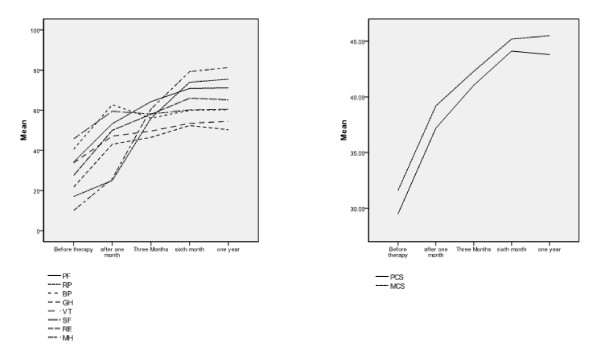
**Mean value of the eight domain scores of the SF-35 v2 on follow-up after stem cell therapy in group I**. BP, bodily pain; group I, SCT group; group II, SMT group; group III, healthy volunteers group; GH, general health; MCS, mental component summary; MH, mental health; PCS, physical component summary; PF, physical functioning; RE, role emotional; RP, role physical; SF, social functioning; VT, vitality.

By following up the SCT group (I) of patients, after stem cell therapy, it was found that both main domains, PCS (physical component summary) and MCS (mental component summary), increased progressively after therapy up to the sixth month of follow up, and then they showed no statistically significant change by the first year after therapy. Still the mean value of the PCS in healthy control group is greater than that of SCT group (I), and this difference is statistically significant, with a *P *value = 0.00. No statistically significant difference was noted between the values of corresponding MCS values between both groups (Figure 2; Table 3).

**Table 3:** The *P *value for comparison of SF-v2 domains in the follow-up period in group (I)

Follow-up periods	*P *value for SF domains									
	
	PCS	MCS	PF	RP	BP	GH	VT	SF	RE	MH
Before therapy versus 1 month after therapy	0.000	0.00	0.00	0.00	0.00	0.00	0.00	0.00	0.00	0.00

1 versus 3 months after therapy	0.000	0.002	0.00	0.00	0.06	0.00	0.14	0.008	0.00	0.49

3 versus 6 months after therapy	0.004	0.004	0.01	0.005	0.14	0.02	0.012	0.008	0.002	0.2

6 months versus 1 year after therapy	0.78	0.69	0.9	0.82	0.93	0.34	0.45	0.814	0.7	0.76

BP, bodily pain; GH, general health; MCS, mental component summary; MH, mental health; PCS, physical component summary; PF, physical functioning; RE, role emotional; RP, role physical; SF, social functioning; VT, vitality.

The same applies for PF, RP, SF, and RE, in which the score increased progressively during the months of follow-up to the sixth month, and then it became stable; no statistically significant difference exists between the scores at the sixth month and those at 1 year, and still the values of both PF and SF in the healthy control group are greater than the corresponding values after 1 year of follow-up in the SCT group (I) of patients with a *P *value = 0.00 in each. No statistically significant difference regarding RP and RE values is apparent at the same periods of follow-up between both groups.

For the BP and MH domains, the issue is a little bit different, the score increased significantly after one month of therapy. No statistically significant changes were noted afterward, and yet it was found that the scores of BP after 1 month of therapy in the SCT group (I) were less than those of healthy control group, and the same applies to the values of MH at the same period. The difference is statistically significant with a *P *value = 0.001, yet these scores are also significantly greater than those of SMT group (II).

The SCT group (I) also reported a significant increase in both GH and VT domain scores after 1 month of therapy; then it insignificantly changed until the third month of follow-up, and then both domains showed a statistically significant increase in their scores by the sixth month, but again, the scores were insignificantly changed. After that, both domains showed no statistically significant changes in their scores after 1 year of follow-up, yet still both scores were significantly greater in the healthy volunteers group than in the SCT group (I).

By following up the patients receiving standard care in the SMT group (II) with the SF-36 v2, we noted a significant deterioration in all the domain scores throughout the follow-up period, which does not match the observed improvement in the scores of patients treated with stem cells, with a statistically significant difference between both groups in all the domains.

**Figure 3:**
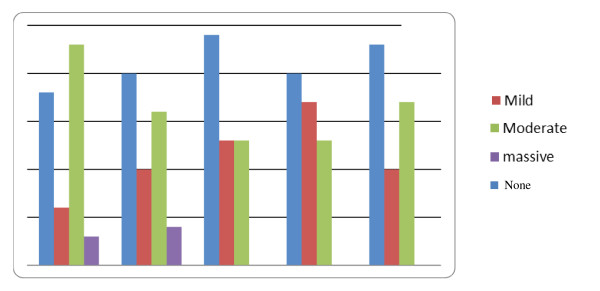
**Progress of the degree of ascites in group (I) during the follow-up period**. Y axis represents the percentage of patients; X-axis represents the time of the follow-up period, and the figure represents the change of the degree of ascites during the follow-up period.

Regarding ascites, patients of the SCT group (I) showed continuous improvement throughout the follow-up period, represented in Figure 3.

To study the effect of diabetes mellitus in the SF-36 questionnaire, the patients of the SCT group (I) were categorized into two groups. The first group was for patients with diabetes mellitus, and the second group was for patients with no diabetes mellitus. The results showed no significant difference between both diabetes and nondiabetes groups regarding the PCS and MCS score before and after therapy; both domains increased significantly after therapy in both groups. Regarding the PCS domain before therapy, both diabetes and nondiabetes groups showed a statistically significant positive relation with PCS after 1 and 3 months of follow-up. The same applies to the MCS score, but the relation extends in the nondiabetes group to show a statistically significant relation between MCS before therapy and that after 6 months of therapy.

**Figure 4:**
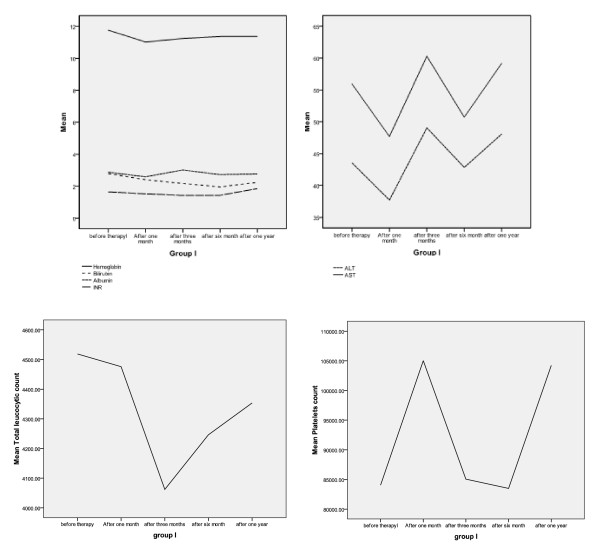
**Changes that occurred in laboratory data during the follow-up period of group I**. In the upper two graphs, the y-axis represents the mean value of the tested parameter, and the x-axis represents the follow-up periods. Upper-left graph shows changes in the mean levels of hemoglobin, total serum bilirubin, INR, and serum albumin, whereas the upper-right graph represents the mean values of both ALT and AST. The lower-left graph represents the mean total leukocyte count; and the lower-right graph represents the mean platelet count.

**Figure 5:**
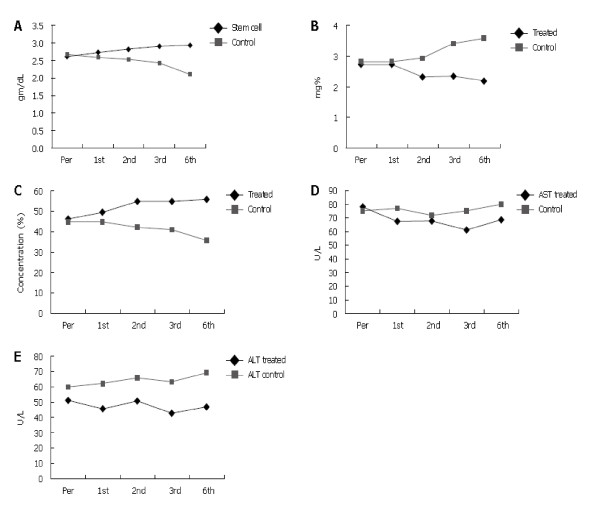
**Follow-up data from our previous study**. Changes in serum albumin **(A)**, serum bilirubin **(B)**, prothrombin concentration **(C)**, aspartate transaminase values **(D)**, alanine transaminase values **(E)**, in both control and treated groups. Pre, pretreatment; 1^st^, first month; 2^nd^, second month; 3^rd^, third month; 6^th^, sixth month after treatment.

The change in the laboratory data throughout the follow-up period in the SCT group (I) is represented in Figure 4 and Table 4, whereas those of our previous study are represented in Figure 5.

**Table 4:** The *P *values of comparison of the laboratory data during the follow-up period in group (I)

Follow-up periods	*P *value for lab data					
	
	Hemoglobin	TLC	Platelets	Bilirubin	ALT	AST
Before therapy versus after 1 month	0.00	0.78	0.19	0.00	0.00	0.00

After 1 month versus after 3 months	0.285	0.06	0.25	0.00	0.00	0.00

After 3 months versus after 6 months	0.15	0.11	0.35	0.002	0.001	0.00

After 6 months versus after 1 year	0.97	0.16	0.303	0.00	0.013	0.00

ALT, alanine transaminase; AST, aspartate transaminase; TLC, total leukocyte count.

In SCT group (I), the PCS domain at 1 year after therapy is dependent on the AST value at the third month after therapy, in which a significant positive relation is seen: *r *= 0.33 with a *P *value of 0.009. The MCS domain after 1 year of therapy is dependent on the ALT value before therapy, in which a significant negative relation was detected with *r *= -0.268 and *P *value = 0.03. The MCS domain after 6 months of therapy is dependent on the AST value before therapy, in which a significant negative relation was detected with *r *= -0.37 and *P *value = 0.048. A significant positive relation was also detected between PCS after 3 months of therapy and the value of ALT before therapy, with *r *= 0.27 and *P *value = 0.03. A significant positive relation was detected between the RP after 1 year of therapy and the value of total bilirubin before therapy, with *r *= 0.302 and *P *value = 0.017. A significant positive relation was detected between RP after 1 year of therapy and the value of ALT after 1 month, with *r *= 0.241 and *P *value = 0.046.

**Figure 6:**
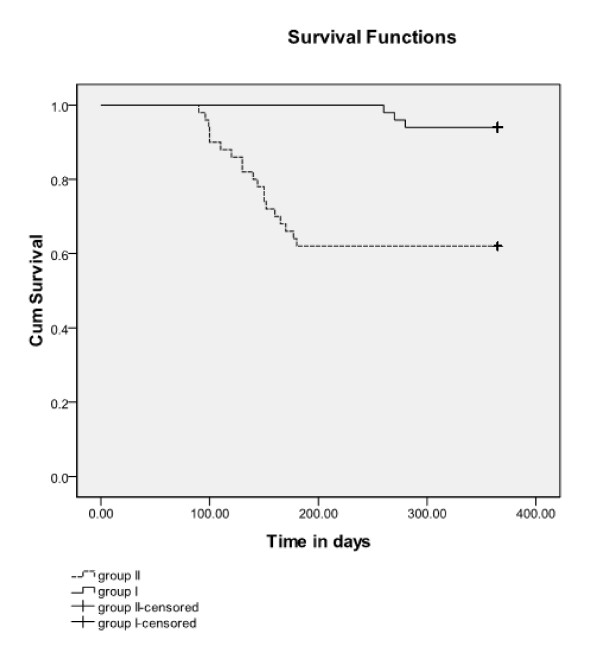
**Time to death over a 1-year period, group I versus group II**. Survival curve, log rank = 0.00.

The overall survival of patients of both groups after 1 year of follow-up was 62% in the SMT group (II) and 94% in the SCT group (I), with a mean survival time of 277.56 days (95% CI, 246.217 to 308.903) for the SMT group (II) and 359.300 days (95% CI, 353.022 to 365.578) for the SCT group (I). Time to death for both groups is represented in the Kaplan-Meier curve with a log rank of 0.00 (Figure 6).

## Discussion

Cell-based therapy, including the use of stem cell therapy, offers considerable hope for patients with end-stage liver cell failure [9,10]. We report a prospective cohort study using a quality-of-life survey, SF-36 v2, conducted on 50 posthepatitis C virus cirrhosis patients receiving stem cell therapy at our center, to be compared with the survey results conducted on a group of posthepatitis C virus cirrhosis patients (*n *= 50), receiving the standard line of care, and a group of healthy blood donors (*n *= 50).

The overall compliance rate for survey completion in our study was high (98%). This rate reflects our policy of monitoring all patients who received stem cell therapy for 1 year at our center and administering the surveys, in person, at those scheduled visits. This policy differs from other quality-of-life reports used in similar studies in liver transplantation patients [11,12], but we believe that this approach provides maximum information regarding the impact of stem cell therapy on cirrhosis patients at various time points.

Our study was conducted in a nonprofit governmental hospital where the patients are of low socioeconomic status; this fact may influence their HRQoL scores and the results of our study as well, which may differ from those with patients attending a private hospital in Egypt.

The pretherapy SF-36 v2 scores of patients in SCT group (I) were significantly below average as compared with those of the healthy control subjects. This is an expected finding because they are patients with end-stage liver disease. In comparison of their scores with those of patients in the SMT group (II), we found that PCS, GH, BP, VT, and MH scores show no statistically significant difference between the groups, but PF is significantly greater in the SCT group (I), whereas MCS, RE, SF, and RP are significantly greater in the SMT group (II).

Our patients found the overall stem cell therapy process positive; this was reported through the consistent improvement of the patients' PCS and MCS scores in all patients until the end of the first year of follow-up to reach the average values of the healthy volunteers, indicating that stem cell therapy improves the HRQoL. Most of the SF-36 eight domains (PF, RP, SF, RE, GH, and VT) showed consistent improvement during the follow-up period in all patients back to pretherapy; this accords with reports by other studies [13,14].

Regarding the laboratory data of patients in SCT group (I), it was noticed that platelets and WBCs (white blood cells) counts did not show any statistically significant change during the follow-up period, but a significant reduction in hemoglobin and albumin levels occurred in the first month of follow-up; hemoglobin reached a steady level until the end of the follow-up period, whereas albumin showed a significant increase by the third month of follow-up, but it decreased significantly again to reach a steady value until the end of the follow-up period. This result coincides with those of some published studies [5,6], but does not agree with other published studies [10,14,15].

INR values continued to decrease significantly until the third month of follow-up, but a marked significant increase was observed by the end of the first year of follow-up, which agrees with what was concluded by Lyra *et al. *[13].

The total serum bilirubin levels continue to decrease up to the sixth month of follow-up. Then they show a significant increase by the end of the first year of follow-up, but significantly less than its pretherapy level. These findings are consistent with the results of other studies [5,8,10,14,16,17].

For the liver enzymes, both AST and ALT levels showed statistically significant changes during the period of follow-up; at the first month after therapy, they decreased, then increased by the third month, and then decreased again by the sixth month, to increase by the end of the first year of follow-up. It worth mentioning that the significant change in the biochemical profile and the ascites grade in the SCT group (I) had markedly decreased the need for receiving the standard line of care for such patients, so the patients were managed accordingly.

In the SCT group (I), in comparing the change in the clinical findings with changes in the quality of life, we noticed that at 1 month, the quality of life scores was improved together with laboratory results; this agrees with that reported by Kondo *et al. *[3]. However, the liver enzymes deteriorated after being improved after 3 months, and the total bilirubin increased again after 6 months, while all the quality-of-life domains continued to improve, except for the BP and MH, which remained almost constant after the first month. This is significantly less than those of the normal healthy subjects, which means that the quality-of-life domains are not affected by the changes in the liver function, except in the early stage after stem cell therapy, which does not agree with results reported by El Garem *et al. *[6]. This may be attributed to the difference in the technique used for stem cell therapy; El Garem injected 5 to 10 million cells in 10 ml under sonographic guidance into the spleens of his patients.

Our result might be explained by the hypothesis that the bodily pain subscale is a two-item scale that measures perception of pain and how much pain interferes with normal work, including both outdoor work and housework, where the score does not continue to improve after the first month and becomes constant. This may be due to mild significant elevation of liver enzymes after the first month; moreover, some patients develop mild to moderate ascites, which leads to abdominal pain in some patients.

Conversely, the mental health subscale is a five-item scale that measures nervousness, happiness, depression, peacefulness, and feeling so down-in-the-dumps. Its score does not continue to increase after the first month, and that may be attributed to the depression and the nervousness that the end-stage liver disease patients experience and directly affects their work; their activity become much less than usual.

The SF-36 v2 is a subjective health survey; our results showed that no close association occurs between subjective and objective data such as liver-function tests, except in the early post-stem therapy phase. These observation agree with those of Kondo *et al. *[3], but on following up our patients, the issue becomes different, and this may be because no follow-up data were acquired in the Kondo study; moreover, this is also attributed to the difference in the studied groups. In Kondo *et al. *[3], the study group was hepatocellular carcinoma patients. Conversely, some significant relations between the SF-36 v2 domain scores and laboratory data were detected in our studied group of patients. A relation exists between SF-36 v2 domains after 1 year of therapy; PCS, MCS, and RP with AST after 3 months of therapy; ALT before therapy, and ALT after 1 month of therapy, respectively. A relation exists between the laboratory data before therapy and AST, ALT, and total bilirubin and MCS after 6 months, PCS after 3 months, and RP after 1 year of therapy.

After 1 year of follow-up of both SCT and SMT group, mortality was reported in three cases (6%) of the SCT group (I), whereas 19 (38%) cases of the SMT group (II), with a statistically significant difference between the mean survival times of both groups and no malignancy cases reported in the SCT group (I) after treatment all through the follow-up period. This difference agrees with what was reported in Nouman *et al. *[8], but with lower mortality rates in the stem cell-treated group of patients.

This study suggests that the SCT patient finds the overall experience to be a positive one. The study also allowed us to critically examine the various aspects of our patient-evaluation process and to develop recommendations for future use. Some of the major suggestions derived from this study are as follows: (a) It should be stressed that significant postoperative pain will occur, but it can be controlled; (2) follow-up at an SCT clinic should be provided for a 1-year period, when possible, to allow the detection of late-occurring complications related to therapy that may go unrecognized in other settings; and (c) donors should be provided with a realistic assessment of what the SCT outcome is likely to be.

A potential weakness in our method of evaluating SCT recipient quality of life is that a member of the SCT team had conducted the surveys by himself. This approach clearly results in a high rate of survey compliance, but it may also deter some patients from answering sensitive questions truthfully, as a clear association between the respondent and answer by the physician is possible. Currently, we are considering providing the donors each with a short questionnaire and a stamped, self-addressed envelope. They will be asked to complete the survey and return it anonymously.

The results of our study showed no influence of diabetes mellitus on the HRQoL scores of patients who were subjected to SCT before therapy or after therapy during the period of follow-up, where the SF-36 v2 domains scores are insignificantly different in diabetic patients than those with no diabetes. This does not agree with the concept represented in 2000 by William H. Polonsky [18]. This may be explained because the diabetes patients in our study were well controlled with either insulin or oral hypoglycemic drugs; moreover, no diabetic complications were encountered in our studied group.

## Conclusions

In contrast to the influence of medical treatment, SCT improves the SF-36 v2 scoring system across time. This improvement is not completely dependent on the changes in the objective clinical variables that occurred during the same follow-up period, raising the question to include a new parameter to reflect the liver condition differently from the commonly used ones. No significant influence of diabetes mellitus on SF-36 v2 scores was detected during the follow-up period, and it is recommended to study the influence of diabetes mellitus on the HRQoL in patients receiving SCT in a larger cohort of patients. Improved survival of patients occurs after SCT.

## Abbreviations

ALP: alkaline phosphatise; ALT: alanine transaminase; AST: aspartate aminotransferase; BP: bodily pain; CBC: complete blood count; CLD: chronic liver disease; CT: computed tomography scan; G-CSF: Neupogen: Roche Pharmaceutical; GH: general health; HCC: hepatocellular carcinoma; HCV: hepatitis C virus; HRQoL: health-related quality of life; INR: prothrombin time and concentration; MCS: mental component summary; MH: mental health; MNC: mononuclear cell; PCS: physical component summary; PF: physical functioning; RBC: red blood cell; RE: Role Emotional; RP: Role Physical; SCT: stem cell therapy; SD: standard deviation; SF-36: The Short Form-36; SF: social functioning; SMT: supportive medical treatment; VT: vitality; WBC: white blood cell.

## Competing interests

The authors declare that they have no competing interests.

## Authors' contributions

HMS shared in the study design and managed the stem cell-treated patients. ARNZ generated the idea and was responsible for the stem cell laboratory, editing, and revising the manuscript. RAA was involved in the acquisition of data from stem cell-treated patients and the control group. IM helped in the management of chronic hepatitis patients and the control group. EA shared in the management of the chronic active hepatitis patients and the control group. TD did the data analysis and interpretation. OSA performed the stem cell isolation and differentiation in the stem cell laboratory. AAB shared in stem cell isolation and differentiation, RT-PCR of differentiated cells, and helped in editing the manuscript. All authors read and approved the manuscript for publication.
